# The coxsackievirus and adenovirus receptor acts as a tumour suppressor in malignant glioma cells

**DOI:** 10.1038/sj.bjc.6600932

**Published:** 2003-04-29

**Authors:** M Kim, L A Sumerel, N Belousova, G R Lyons, D E Carey, V Krasnykh, J T Douglas

**Affiliations:** 1 Division of Human Gene Therapy, Departments of Medicine, Pathology and Surgery, and the Gene Therapy Center, University of Alabama at Birmingham, Birmingham, AL 35294, USA; 2 Medical Statistics Section, Division of Hematology and Oncology, Department of Medicine, University of Alabama at Birmingham, Birmingham, AL 35294, USA

**Keywords:** CAR, glioma, tumour suppressor

## Abstract

The coxsackievirus and adenovirus receptor (CAR) is a membrane glycoprotein with a cytoplasmic domain, a transmembrane domain and an extracellular region consisting of two immunoglobulin-like domains, an amino-terminal immunoglobulin variable (IgV)-related domain (D1), which is distal to the cell surface, and a proximal IgC2 domain (D2). The coxsackievirus and adenovirus receptor has been shown to exhibit tumour suppression activity in human bladder and prostate cancer cells. In the current paper, we demonstrate that CAR is a tumour suppressor in glioma cells and that the extracellular D2 domain is not required for this inhibitory effect. This finding provides a biological basis for the observation that expression of CAR is downregulated in malignant glioma cells. This suggests that strategies to redirect adenoviruses to achieve CAR-independent infection will be necessary to realise the full potential of adenoviral vectors for cancer gene therapy.

The first step in infection by many human adenoviruses, including the subgroup C serotypes 2 and 5 most commonly used in gene therapy applications, is the high-affinity binding of the knob domain of the fibre protein (Henry *et al*, 1994; Louis *et al*, 1994) to the primary cellular receptor, the coxsackievirus and adenovirus receptor (CAR). The coxsackievirus and adenovirus receptor is a 46 kDa class I membrane glycoprotein with a carboxy-terminal cytoplasmic domain, a transmembrane domain and an extracellular region consisting of two immunoglobulin-like domains, an amino-terminal immunoglobulin variable (IgV)-related domain (D1), which is distal to the cell surface, and a proximal IgC2 domain (D2). Although there is evidence for the evolutionary conservation of CAR, with homologues of the human receptor, hCAR, present in several mammalian species, including mice (Tomko *et al*, 1997; Bergelson *et al*, 1998), rats, dogs and pigs (Fechner *et al*, 1999) as well as in zebrafish (van Raaij *et al*, 2000), the normal physiological function of this membrane protein is unknown.

The identification of CAR as the primary adenovirus receptor triggered numerous studies demonstrating that primary cancer cells are frequently refractory to adenoviral infection because of a paucity of CAR on the cell surface (Dmitriev *et al*, 1998; Hemmi *et al*, 1998; Miller *et al*, 1998; Blackwell *et al*, 1999; Li *et al*, 1999; Vanderkwaak *et al*, 1999). More recently, it has been shown that the expression of CAR in highly tumorigenic CAR-deficient human prostate and bladder cancer cells leads to a growth-inhibitory effect (Okegawa *et al*, 2000,^2001^). We have previously reported that the tumorigenic human U-118 MG glioblastoma cell line is refractory to adenoviral infection because of a paucity of CAR although it expresses the *α*_v_ integrins necessary for virus internalisation (Miller *et al*, 1998). In the current study, we have tested the hypothesis that CAR serves as a tumour suppressor in this cell type. We also sought to determine which structural domains of CAR are responsible for its tumour-suppressive activity.

## MATERIALS AND METHODS

### Construction of plasmids encoding mutant forms of CAR

The 2.4 kb *Bam*HI–*Not*I fragment carrying the hCAR cDNA was first subcloned from pcDNAI.hCAR (obtained from Robert W Finberg, Harvard Medical School, Boston, MA, USA) into the mammalian expression vector pcDNA3 (Invitrogen, Carlsbad, CA, USA) to give pcDNA3-hCAR. This plasmid was then used as template for PCR mutagenesis to construct the tailless hCAR truncation mutant by insertion of a stop codon after the position corresponding to amino-acid residue 260 (Wang and Bergelson, 1999). This construct therefore contained the extracellular domain, transmembrane domain and the first two amino acids from the cytoplasmic domain of hCAR. The hCAR-GPI construct was generated by overlap extension PCR to fuse the extracellular domain of hCAR (corresponding to amino-acid residues 1–235) to the 37 carboxy-terminal amino acids of human decay-accelerating factor (DAF) (Wang and Bergelson, 1999). The PCR products were inserted into the *Bam*HI and *Not*I sites of pcDNA3 to give pcDNA3-hCAR-tailless and pcDNA3-hCAR-GPI, respectively. Deletion mutants of hCAR lacking the D1 or D2 extracellular domains (lacking amino acids 21–144 and 145–233, respectively (Freimuth *et al*, 1999)) were constructed by overlap extension PCR using pcDNA3-hCAR as the template. The PCR products were inserted into the *Bam*HI and *Not*I sites of pcDNA3 to give pcDNA3-hCARΔD1 and pcDNA3-hCARΔD2, respectively. The integrity of all constructs was verified by DNA sequencing using the CEQ2000XL DNA analysis system (Beckman-Coulter, Fullerton, CA, USA).

### Cell culture

CAR-positive 293 cells (Graham *et al*, 1977) were purchased from Microbix (Toronto, Ontario, Canada) and CAR-negative human U-118 MG glioma cells were obtained from the American Type Culture Collection (Manassas, VA, USA). The cells were propagated at 37°C in a 5% CO_2_ atmosphere in 50 : 50 mixture of Dulbecco's modified Eagle's medium and Ham's F-12 medium (DMEM/F-12), supplemented with 10% (v v^−1^) fetal calf serum (FCS), L-glutamine (2 mM), penicillin (100 U ml^−1^) and streptomycin (100 *μ*g ml^−1^). Stable transfectants were maintained in 400 *μ*g ml^−1^ G418. FCS was purchased from Gibco-BRL (Grand Island, NY, USA), and media and supplements were from Mediatech (Herndon, VA, USA).

### Transfection of U-118 MG cells and selection of stable clones

U-118 MG cells were transfected with the plasmids encoding hCAR or the hCAR deletion mutants using Superfect (Qiagen, Valencia, CA, USA) according to the manufacturer's protocol. Cells stably expressing hCAR or the hCAR deletion mutants were selected in the presence of G418 at a concentration of 400 *μ*g ml^−1^.

#### Flow cytometry

Confluent cells were released with Versene and resuspended in phosphate-buffered saline (PBS; Mediatech) containing 1% bovine serum albumin (BSA; Boehringer Mannheim, Indianapolis, IN, USA) at a concentration of 1–2 × 10^6^ cells per ml. One hundred microliters of cells (1–2 × 10^5^) were incubated with 100 *μ*l of anti-hCAR monoclonal antibody RmcB at a concentration of 10 *μ*g ml^−1^ for 1 h at 4°C with constant shaking. Cells were then washed with PBS containing 1% BSA and incubated with 100 *μ*l of fluorescein isothiocyanate (FITC)-labelled rabbit anti-mouse IgG, Fc-specific secondary antibody (Jackson Immunoresearch Laboratories,West Grove, PA, USA) at a concentration of 10 *μ*l ml^−1^ for 1 h at 4°C with constant shaking. Cells were washed with PBS containing 1% BSA and fixed to a final volume of 300 *μ*l of 1–2% paraformaldehyde. Samples were then analysed by flow cytometry in the University of Alabama FACS Core Facility on an FACSCalibur machine using Cell quest FACS analysis software (Becton-Dickinson, Franklin Lakes, NJ, USA).

### Adenoviral vectors

Ad5Luc1 is a first generation, E1-, E3-deleted Ad5 vector, which expresses firefly luciferase under the control of the cytomegalovirus (CMV) immediate-early promoter (Krasnykh *et al*, 2001). The recombinant adenoviral vectors were propagated on the permissive 293 cell line, purified by two rounds of cesium chloride density centrifugation and plaque titred on 293 cells according to standard techniques (Anon, 1998).

### Adenovirus-mediated reporter gene transfer assays

In experiments to assay adenovirus-mediated luciferase gene delivery, 70% confluent U-118 MG cells or the stable transfectants in a 24-well tray were infected at a multiplicity of infection (MOI) of 100 plaque-forming units (PFU) per cell by adding Ad5Luc1 diluted in 100 *μ*l DMEM+2% FCS and incubating for 30 min at room temperature. The unbound virus was then aspirated and the cells were washed with DMEM+2% FCS and incubated in 100 *μ*l DMEM+2% FCS for 1 h at 37°C. One hundred microlitres of DMEM+10% FCS were then added and the cells were incubated at 37°C for a further 24 h to allow luciferase expression. The cells were then lysed and assayed for luciferase activity using a luciferase assay system (Promega, Madison, WI, USA) according to the manufacturer's protocol.

### *In vitro* soft agar colony-formation assay

U-118 MG cells were transfected with the various hCAR expression vectors and stable transfectants selected in the presence of 400 *μ*g ml^−1^ G418. Monolayers of cells were released with Versene and dispersed into a suspension of single cells in growth medium. One thousand cells were resuspended in 2 ml of growth medium containing 0.4% low melting temperature agarose (Sea Plaque; FMC Products) and 400 *μ*g ml^−1^ G418, and were overlaid in triplicate on 2 ml of solidified 0.8% low melting temperature agarose in growth medium in a 6-well dish. The dishes were incubated for 18 days at 37°C in a 5% CO_2_ atmosphere. Colonies larger than 50 cells were then counted. Descriptive statistics (mean and standard deviation) on colony sizes were calculated. The mean colony sizes±standard deviations are shown. Statistical analysis was performed using one-way ANOVA. *P*<0.05 was considered statistically significant. All statistical tests were performed with Statistical Analysis Software (Version 6.12; SAS Institute, Inc., Cary, NC, USA).

### *In vivo* tumorigenicity assay

Tumour xenografts were established by subcutaneous injection of 5 × 10^6^ U-118 MG cells or the stably transfected derivatives into the flank of 8- to 10-week-old female athymic nude mice (nine or 10 mice per group) (*nu*/*nu*; Frederick Cancer Center, Frederick, MD, USA). Bidimensional tumour measurements were taken with calipers and the tumour volume was calculated using the simplified formula for a rotational ellipsoid: 0.5 × length × width^2^ (Dethlefsen *et al*, 1968). Statistical analysis was performed by one-way ANOVA using SAS. *P*<0.05 was considered statistically significant.

## RESULTS

**Figure 1:**
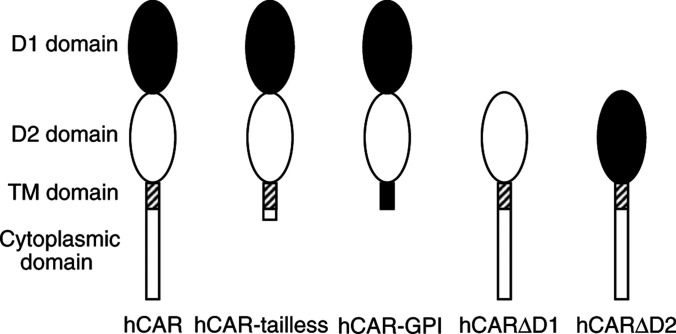
Schematic diagram of hCAR and deletion mutants. The coxsackievirus and adenovirus receptor comprises an extracellular region, a transmembrane domain (hatched rectangle) and a cytoplasmic domain (open rectangle). The extracellular region of wild-type CAR comprises an amino-terminal IgV-related domain designated D1 (solid oval), which is distal to the cell surface, and a proximal IgC2 domain designated D2 (open oval). The hCAR-tailless mutant is truncated after amino acid 260. The hCAR-GPI mutant consists of the extracellular domain of hCAR (corresponding to amino-acid residues 1–235) fused to the 37 carboxy-terminal amino acids of human DAF. The hCARΔD1 mutant lacks amino acids 21–144, while the hCARΔD2 mutant lacks amino acids 145–233 (Freimuth *et al*, 1999).

We first constructed two previously described truncation mutant forms of hCAR–tailless hCAR, containing the extracellular domain, transmembrane domain and the first two amino acids from the cytoplasmic domain of hCAR, and glycosylphosphatidyl (GPI)-anchored hCAR, lacking both the transmembrane and cytoplasmic domains (Figure 1

) (Wang and Bergelson, 1999). The hCAR cDNA was first subcloned into the mammalian expression vector pcDNA3 to give pcDNA3-hCAR. This plasmid was then used as template for PCR mutagenesis to construct the tailless hCAR truncation mutant by insertion of a stop codon after the position corresponding to amino-acid residue 260 (Wang and Bergelson, 1999). The CAR-GPI construct was generated by overlap extension PCR to fuse the extracellular domain of hCAR (corresponding to amino-acid residues 1–235) to the 37 carboxy-terminal amino acids of human decay-accelerating factor (DAF) (Wang and Bergelson, 1999). The PCR products were inserted into pcDNA3 to give pcDNA3-hCAR-tailless and pcDNA3-hCAR-GPI, respectively. The integrity of the constructs was verified by DNA sequencing.

**Figure 2:**
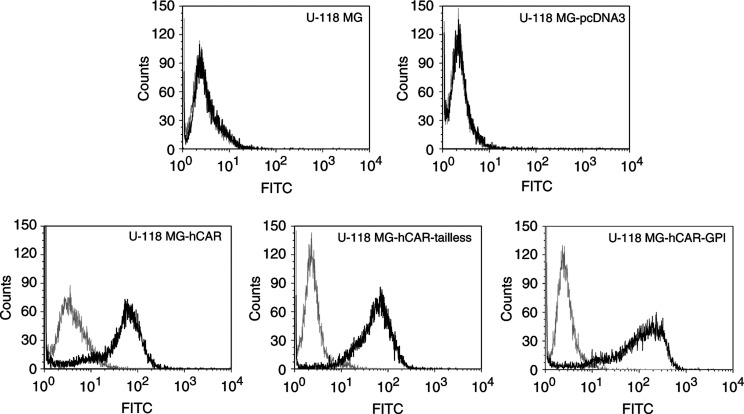
FACS analysis of stably transfected cells to confirm surface expression of hCAR and deletion mutants. Dark line is anti-hCAR primary monoclonal antibody RmcB plus FITC-labelled secondary antibody. Light line is FITC-labelled secondary antibody in the absence of primary antibody.

Monolayers of CAR-negative human U-118 MG malignant glioma cells were stably transfected with pcDNA3-hCAR, pcDNA3-hCAR-tailless, pcDNA3-hCAR-GPI or with the parental pcDNA3 plasmid as a control. Individual single-cell clones were isolated and expanded by selection in the presence of 400 *μ*g ml^−1^ G418. Clones expressing equivalent levels of hCAR and the truncation mutants were identified on the basis of their susceptibility to infection by Ad5Luc1 (Krasnykh *et al*, 2001), an E1-deleted Ad5 vector which expresses the luciferase reporter gene under the control of the CMV promoter (data not shown). Expression of hCAR and the truncation mutants on the surface of the cells was confirmed by fluorescence-activated cell sorting (FACS) analysis using anti-hCAR monoclonal antibody RmcB, which verified that the selected clones represented pure populations of stably transfected cells (Figure 2

).

**Figure 3:**
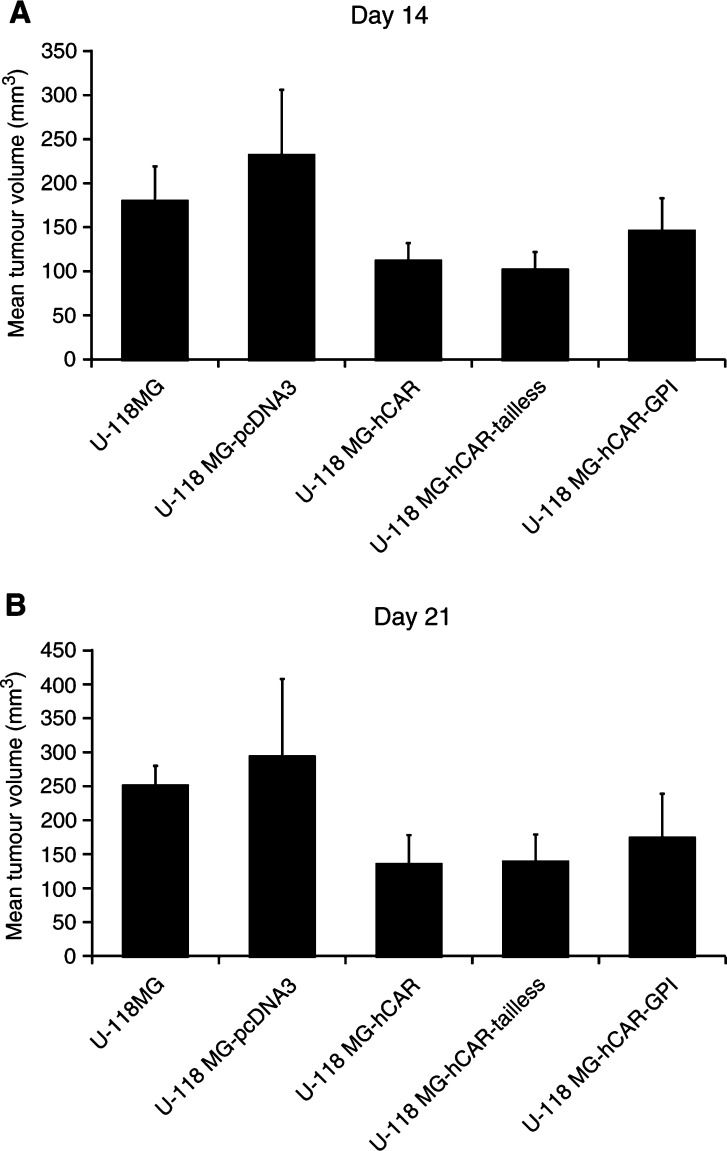
*In vivo* tumorigenicity assay. Tumour xenografts were established by subcutaneous injection of 5 × 10^6^ U-118 MG cells or the stably transfected derivatives into the flank of 8- to 10-week-old female athymic *nu*/*nu* nude mice (nine mice per group). Bidimensional tumour measurements were taken with calipers and the tumour volume was calculated using the simplified formula for a rotational ellipsoid: 0.5 × length × width^2^ (Dethlefsen *et al*, 1968). The mean tumour volumes±standard deviations on days 14 (**A**) and 21 (**B**) are shown. Statistical significance was achieved if *P*<0.05, based upon a one-way ANOVA.

Tumour xenografts were established by subcutaneous injection of 5 × 10^6^ U-118 MG cells or the stably transfected derivatives into the flank of 8- to 10-week-old female athymic nude mice (nine mice per group). Bidimensional tumour measurements were taken with calipers and the tumour volume was calculated. The mean tumour volumes on days 14 and 21 are shown in Figure 3

. As expected, stable transfection of the U-118 MG cells with the parental pcDNA3 vector did not inhibit the growth of the subcutaneous tumors (*P*>0.05 on days 14 and 21). However, the U-118 MG cells stably transfected with hCAR showed a significant decrease in tumorigenicity relative to the parental cells (*P*<0.05 on days 14 and 21, respectively). The U-118 MG cells stably transfected with hCAR-tailless likewise showed a significant decrease in tumorigenicity relative to the parental cells (*P*<0.01 on day 14; *P*<0.05 on day 21), whereas no inhibition in tumour growth was observed with the cells stably transfected with hCAR-GPI (*P*>0.05 on days 14 and 21). These findings are in accordance with earlier reports that the extracellular and transmembrane domains of hCAR, together with the first two amino acids of the cytoplasmic domain, are required for its biological activity in suppressing growth in human prostate and bladder cancer cells (Okegawa *et al*, 2000,^2001^).

We then sought to examine the role of the extracellular domains of hCAR in the tumour-suppressive activity. Deletion mutants of hCAR (Figure 1) lacking the D1 or D2 extracellular domains (lacking amino acids 21–144 and 145–233, respectively (Freimuth *et al*, 1999)) were constructed by overlap extension PCR using pcDNA3-hCAR as the template. The PCR products were subcloned into pcDNA3 to give pcDNA3-hCARΔD1 and pcDNA3-hCARΔD2, respectively. The integrity of the constructs was verified by DNA sequencing.

**Figure 4:**
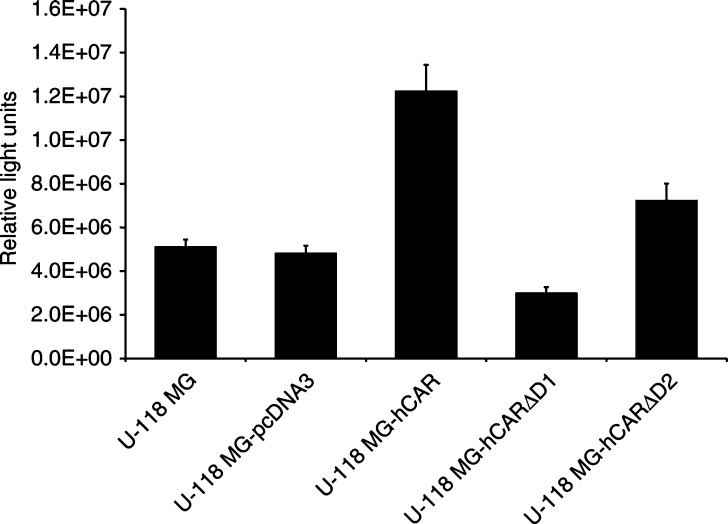
Adenovirus-mediated gene transfer to U-118 MG and derivative cells. Monolayers of CAR-negative human U-118 MG malignant glioma cells were transiently transfected with pcDNA3-hCAR, pcDNA3-hCARΔD1 and pcDNA3-hCARΔD2. Forty-eight hours post-transfection, the cells were infected at a multiplicity of infection of 100 p.f.u. per cell with Ad5Luc1. After incubation for 1 h at 37°C, the vector was aspirated and the cells incubated at 37°C for 24 h. The cells were then lysed and assayed for luciferase activity, which is expressed as relative light units. Data are reported as the means±standard deviations of triplicate determinations from a representative of three independent experiments. *P*<0.05 was considered statistically significant.

We first investigated the ability of the deletion mutants to function as primary cellular receptors for human adenovirus serotype 5 (Ad5). Monolayers of U-118 MG malignant glioma cells were transiently transfected with pcDNA3-hCAR, pcDNA3-hCARΔD1 and pcDNA3-hCARΔD2. Forty-eight hours post-transfection, the cells were infected at a multiplicity of infection of 100 plaque-forming units per cell with Ad5Luc1. The cells were incubated for a further 24 h and the luciferase activity was determined. As shown in Figure 4

, the parental U-118 MG cells were refractory to adenoviral infection, while expression of full-length CAR rendered the U-118 MG cells susceptible to adenoviral infection. Luciferase expression in the cells transiently transfected with pcDNA3-hCARΔD1 was not enhanced over the parental cells, indicating that the hCAR deletion mutant lacking the membrane-distal extracellular D1 domain was unable to mediate adenoviral infection. In contrast, luciferase activity could be detected in the cells transiently transfected with pcDNA3-hCARΔD2, the plasmid expressing the hCAR deletion mutant lacking the extracellular D2 domain. This indicates that the membrane-displayed D1 domain of CAR was able to serve as a primary receptor for human adenovirus serotype 5. This observation is in accordance with previous studies which have shown that the isolated recombinant CAR D1 domain binds to the adenovirus fibre knob (Freimuth *et al*, 1999).

U-118 MG cells were then stably transfected with the various hCAR expression vectors and individual single-cell clones were isolated and expanded by selection in the presence of 400 *μ*g ml^−1^ G418. Real-time quantitative RT–PCR and immunoblot analysis employing anti-hCAR monoclonal antibodies was performed in an attempt to choose clones expressing equivalent levels of hCAR, hCARΔD1, and hCARΔD2 mRNA and protein. The specificity of these assays for hCAR was assured by employing hCAR-positive 293 cells and hCAR-negative U-118 MG cells as controls. We were able to identify clones expressing approximately the same amounts of hCAR and hCARΔD2, as determined from the respective mRNA and protein levels (data not shown). However, in spite of multiple attempts, we were unable to isolate a clone in which the level of hCARΔD1 mRNA or protein was greater than 10% of the level achievable with hCAR or hCARΔD2 (data not shown).

**Figure 5:**
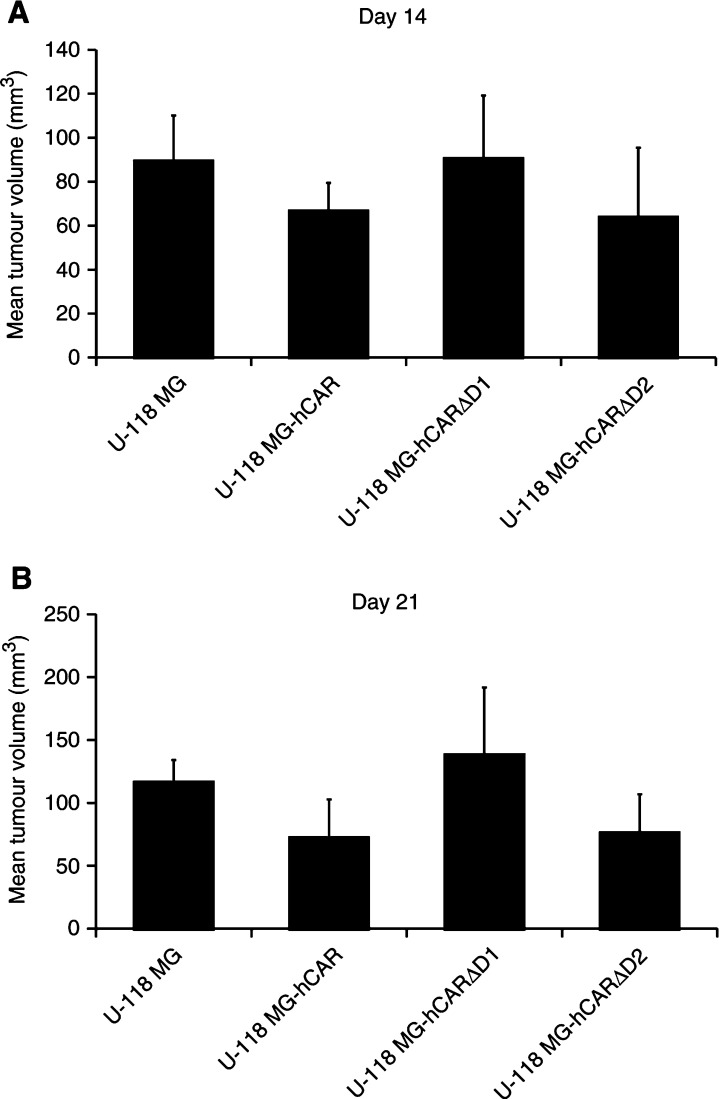
*In vivo* tumorigenicity assay. Tumour xenografts were established by subcutaneous injection of 5 × 10^6^ U-118 MG cells or the stably transfected derivatives into the flank of 8- to 10-week-old female athymic *nu*/*nu* nude mice (10 mice per group). Bidimensional tumour measurements were taken with calipers and the tumour volume was calculated using the simplified formula for a rotational ellipsoid: 0.5 × length × width^2^ (Dethlefsen *et al*, 1968). The mean tumour volumes±standard deviations on days 14 (**A**) and 21 (**B**) are shown. Statistical analysis was performed by one-way ANOVA. *P*<0.05 was considered statistically significant.

Tumour xenografts were again established by subcutaneous injection of 5 × 10^6^ U-118 MG cells or the stably transfected derivatives into the flank of 8- to 10-week-old female athymic nude mice (10 mice per group). The mean tumour volumes on days 14 and 21 are shown in Figure 5

. The U-118 MG cells stably transfected with hCAR showed a significant decrease in tumorigenicity relative to the parental cells (*P*<0.05 on day 14; *P*<0.01 on day 21). Expression of hCARΔD2 also resulted in a significant inhibitory effect on the growth of the U-118 MG tumours (*P*<0.05 on days 14 and 21, respectively). Moreover, the size of the U-118 MG-hCARΔD2 tumours was not significantly different from that of the U-118 MG-hCAR tumours (*P*>0.05 on days 14 and 21, respectively). These results therefore indicate that the expression of hCAR or hCARΔD2 inhibits the tumorigenicity of U-118 MG glioma cells *in vivo*. In contrast, expression of hCARΔD1 did not have a tumour-suppressive effect: the U-118 MG-hCARΔD1 tumours were not significantly different in size from the parental U-118 MG tumours (*P*>0.05 on days 14 and 21), although this could have been due to the low level of expression of hCARΔD1 in the stably transfected U-118 MG cells.

In order to rule out the possibility of the tumour-suppressive activity observed *in vivo* being due to a clonal effect, U-118 MG cells were again transfected with pcDNA3-hCAR, pcDNA3-hCARΔD1 or pcDNA3-hCARΔD2 and stable transfectants selected in the presence of 400 *μ*g ml^−1^ G418. The tumorigenicity of the parental U118-MG cells and pooled clones of the derivative cell lines was determined *in vitro* in a soft agar colony-formation assay. Monolayers of cells were released with Versene and dispersed into a suspension of single cells. One thousand cells were resuspended in 2 ml of growth medium containing 0.4% low melting temperature agarose plus 400 *μ*g ml^−1^ G418 in the case of the transfected cells, and were overlaid in triplicate on 2 ml of solidified 0.8% low melting temperature agarose in growth medium in a six-well dish. The dishes were incubated for 18 days at 37°C in a 5% CO_2_ atmosphere. Colonies containing more than 50 cells were then counted.

**Figure 6:**
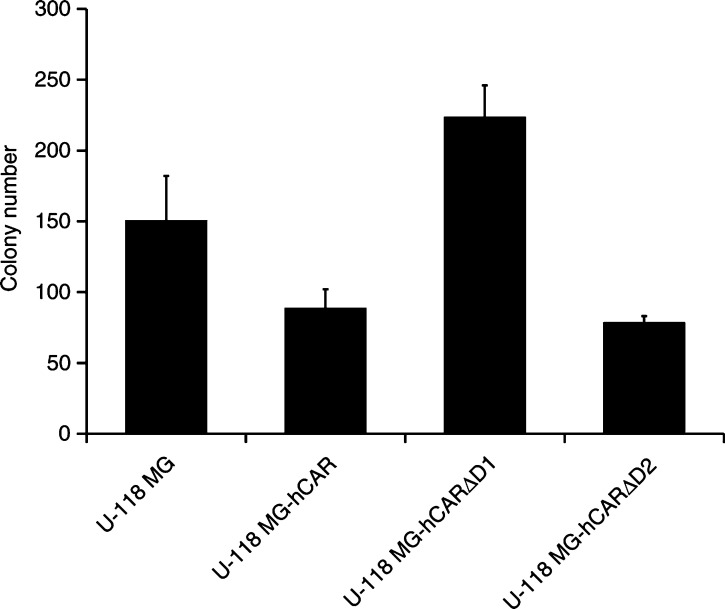
*In vitro* soft agar colony-formation assay. U-118 MG cells were transfected with the various hCAR expression vectors and stable transfectants selected in the presence of 400 *μ*g ml^−1^ G418. Monolayers of cells were released with Versene and dispersed into a suspension of single cells in growth medium. One thousand cells were resuspended in 2 ml of growth medium containing 0.4% low melting temperature agarose (and 400 *μ*g ml^−1^ G418 in the case of the transfectants), and were overlaid in triplicate on 2 ml of solidified 0.8% low melting temperature agarose in growth medium in a 6-well dish. The dishes were incubated for 18 days at 37°C in a 5% CO_2_ atmosphere. Colonies larger than 50 cells were then counted. The mean colony sizes±standard deviations are shown. Statistical analysis was performed by one-way ANOVA. *P*<0.05 was considered statistically significant.

As shown in Figure 6

, expression of hCAR by U-118 MG cells caused a significant reduction in the number of colonies formed by the glioma cells in this assay (*P*<0.05). The mean number of U-118 MG colonies was 149±32, while the mean number of U-118 MG-hCAR colonies was 88±14. The mean number of U-118 MG-hCARΔD2 colonies was 78±5, which was significantly less than the number of colonies formed by the parental U-118 MG cells (*P*<0.05), but not significantly different from the number of U-118 MG-hCAR colonies (*P*>0.05). The mean number of U-118 MG-hCARΔD1 colonies was 223±22, which was significantly greater than the numbers of both U-118 MG-hCAR and U-118 MG-hCAR*Δ*D2 colonies (*P*<0.001 in each case). The results of this soft agar colony-formation assay therefore indicate that expression of both hCAR and hCARΔD2 results in a tumour-inhibitory effect *in vitro*, while expression of hCARΔD1 does not suppress the growth of the glioma cells *in vitro*.

## DISCUSSION

Previous reports have shown that the extracellular and transmembrane domains of hCAR, together with the first two amino acids of the cytoplasmic domain, are required for its biological activity in suppressing growth in human prostate and bladder cancer cells (Okegawa *et al*, 2000,^2001^). We have extended those studies by showing that the extracellular D2 domain of hCAR is not required for CAR to function as a tumour suppressor.

Our findings demonstrate that CAR serves as a tumour suppressor in glioma cells both *in vitro* and *in vivo*. This provides a physiological explanation for the downregulation of CAR observed in malignant glioma cells (Miller *et al*, 1998), although the biological trigger underlying this step in the pathway of malignant transformation remains to be elucidated. Downregulation of CAR has similarly been observed in other tumour types, including ovarian cancer (Dmitriev *et al*, 1998), melanoma (Hemmi *et al*, 1998) and head and neck cancer (Dmitriev *et al*, 1998; Hemmi *et al*, 1998; Li *et al*, 1999). The level of expression of CAR mRNA in bladder cancer specimens from human patients has been shown to correlate inversely with the aggressiveness of the tumour: invasive bladder cancer specimens had significantly reduced CAR mRNA levels compared with superficial bladder cancer specimens (Okegawa *et al*, 2001). Similarly, when compared with normal prostate, CAR expression is decreased in prostate carcinoma specimens of all Gleason scores (Rauen *et al*, 2002). Conversely, metastatic prostate specimens express CAR (Rauen *et al*, 2002). These results suggest that strategies to redirect adenoviruses to achieve CAR-independent infection will be necessary to realise the full potential of adenoviral vectors for gene therapy of primary tumours in the clinical setting (Barnett *et al*, 2002).
